# Precision Medicine in Soft Tissue Sarcoma Treatment

**DOI:** 10.3390/cancers12010221

**Published:** 2020-01-16

**Authors:** Kenji Nakano, Shunji Takahashi

**Affiliations:** Department of Medical Oncology, Cancer Institute Hospital of the Japanese Foundation for Cancer Research, Tokyo 135-8550, Japan; s.takahashi-chemotherapy@jfcr.or.jp

**Keywords:** soft tissue sarcoma, molecular targeted therapy, precision medicine, whole genome sequencing

## Abstract

Soft tissue sarcoma (STS) is a rare component of malignant diseases. STS includes various histological subtypes, and there are some important differences among the different histological subtypes regarding the mutation profile and sensitivity to antitumor agents. Many clinical trials of STS incorporating many different histological subtypes in various populations have been conducted; it is difficult to compare the findings and make conclusions about clinical efficacy. Targeted therapies focusing on specific histological subtypes and precision therapy focusing on the specific genetic mutation(s) of each STS patient are being investigated. Since STS patients are a small population, new clinical trial designs are required to evaluate and establish new targeted therapies for each histological subtype that has a limited number of patients, and preclinical investigations are needed to detect targetable mutations. Now that cancer genome profiling is used in clinical practice, it is urgently necessary to connect the genome profiling data obtained in clinical settings to the optimal clinical treatment strategies. Herein we review the development and challenges of precision therapy in the management of STS patients.

## 1. Introduction

Soft tissue sarcoma (STS) accounts for only 1% of all solid malignant tumors, but it has more than 50 histological subtypes whose clinical and pathological features are diverse and heterogeneous [1]. Because of the rarity and diversity of the STS subtypes, it has been difficult to obtain high-level clinical evidence in large randomized clinical trials of STS patients. Efforts have been made to incorporate as many STS patients as possible in studies in order to gain the necessary clinical evidence, but many clinical trials of STS tended to enroll various histological subtypes in one cohort, until recently. That approach has shown limitations in part because of both the many negative results of these trials and the progress in pathological diagnosis and classification [2]. The new, detailed knowledge about the diversity and heterogeneity of the histological subtypes of STS has evoked the recognition that evaluating various STS histological subtypes in one group of patients would be similar to evaluating solid tumors of different origins (e.g., breast cancer, colorectal cancer, lung cancer and prostate cancer) in one group.

In addition to the diversity of histological subtypes, STS originates from all over the body. About one-half of STSs originate from the extremities and are surgically treated by orthopedic surgeons, but the other half of STS patients have primary disease in one or more gastrointestinal organs, gynecological organs, urological organs, the head and neck, or other sites. Thus, in the localized setting, patients with different primary organs affected by STS are surgically treated by surgeons with different specialties. Both pathological and anatomical factors have made it difficult to perform large-scale STS clinical trials.

Precision medicine in cancer therapy has developed based on advances in both diagnostic technology and molecular targeted therapy and by whole genome sequencing in daily clinical practice, enabling the selection of the appropriate therapy for individual patients [3]. Regardless of the epidemiology and frequency of each tumor, the importance of personalized n-of-1 trials could overcome the current clinical evidence established by large-scale randomized clinical trials [4]. This paradigm shift might help produce new treatment strategies for rare and heterogeneous diseases such as STS.

In this article, we review the history of STS clinical trials, focusing on the changes in patient enrollment from incorporating many patients with various histological subtypes in order to secure the number of patients that is sufficient for the necessary statistical power, to narrowing the patient population for the investigations of precise targeted therapies.

## 2. The Limitations of Incorporating STS Patients Regardless of Their Histological Subtypes

The standard first-line chemotherapy administered to recurrent/metastatic STS patients has been doxorubicin monotherapy for most of the histological subtypes, though there are some exceptions [5]. The addition of another antitumor drug to doxorubicin has been evaluated in several clinical trials, and some of the combinations showed higher overall response rates, but none of combination therapies succeeded in showing superiority to doxorubicin monotherapy in terms of overall survival [6,7,8,9] (Table 1).

In particular, the negative result in the ANNOUNCE trial—a phase 3 trial evaluating the efficacy of the anti-platelet-derived growth factor receptor (PDGFR) antibody olaratumab with doxorubicin—had a significant impact, because olaratumab had already been approved by the U.S. Food and Drug Administration (FDA) as a breakthrough therapy based on the results of a randomized phase 2 trial that showed OS prolongation compared to doxorubicin monotherapy; the European Medicines Agency (EMA) subsequently approved olaratumab [10]. After olaratumab failed to show an overall survival (OS) benefit by the phase 3 ANNOUNCE trial, a negative opinion about conducting clinical trials of STS with different histological subtypes has been dominant [11].

Other than doxorubicin, many antitumor cytotoxic agents have been approved or prescribed with off-label use to STS patients (e.g., ifosfamide, dacarbazine, gemcitabine, and docetaxel), but the clinical evidence regarding these drugs is limited, and there is no evidence that these drugs are superior to doxorubicin. Of them, ifosfamide has been used for many years as monotherapy or in combination with doxorubicin to treat STS, but in a phase 3 trial ifosfamide-based chemotherapies did not show superiority to doxorubicin monotherapy [6,12]. A combination of gemcitabine and docetaxel resulted in a high response rate in a single-arm phase trial, but there are no randomized trials that showed survival benefit. The GeDDiS trial, which compared a combination of gemcitabine and docetaxel to doxorubicin monotherapy, did not show superiority of gemcitabine and docetaxel to doxorubicin monotherapy, and this combination regimen was relatively more toxic than doxorubicin [13]. Another problem is that these candidate drugs were used in different doses and schedules in different clinical trials and clinical reports. There are no established standard doses and schedules in STS treatments.

Considering the above-cited results of clinical trials and discussions, it appears that in STS there is a very low chance of emerging systemic treatments replacing doxorubicin for STS, at least based on the evidence of past and future large-scale randomized clinical trials enrolling various histological subtypes.

## 3. Histology-Based Chemotherapy Investigations Based on Clinical Data

The differences in the sensitivity of STSs to systemic chemotherapy among the histological subtypes of STS and the various antitumor drugs have been discussed for many years. Some of the histological subtypes of STS, most of which were observed in pediatric and young patients, were highly sensitive to systemic chemotherapies. For these subtypes (e.g., Ewing sarcoma and rhabdomyosarcoma), intensive multidrug combination chemotherapy has been incorporated in the standard treatment strategies [14,15], and clinical trials have been designed and performed separately.

In most of the other histological subtypes of STS, doxorubicin-based chemotherapy remains the standard chemotherapy as noted above. However, clinical reports indicated that specific histological STS subtypes were sensitive to specific antitumor drugs other than doxorubicin. The evidence about the underlying mechanisms of action of the other drugs is insufficient, however. For example, synovial sarcoma, which is known as the characteristic chromosomal translocation of t(X;18)(p11.2;q11.2) [16], is sensitive to ifosfamide, and ifosfamide-containing regimens are preferably prescribed for synovial sarcoma patients in clinical practice [17,18], even though there have been no results of prospective randomized clinical trials. The combination of gemcitabine and docetaxel used in the phase 3 trial described above was first examined in a phase 2 trial of leiomyosarcoma, which included a high percentage of cases of uterine origin [19]. The high response rate observed in that phase 2 trial (53%; 15 partial responses of 34 patients) resulted in a tendency for the combination of gemcitabine and docetaxel to be used for leiomyosarcomas (especially those of uterine origin) in clinical practice, despite the negative results of phase 3 or other randomized clinical trials.

Paclitaxel has shown a modest response rate in angiosarcoma, as has doxorubicin-based chemotherapy, but the clinical evidence is limited [20,21]. The majority of cutaneous angiosarcomas (which usually originate from the head and neck skin) has been documented in older individuals, and thus a paclitaxel-based regimen is preferred in clinical practice due to its low risk of cardiotoxicity.

In light of the differences in clinical responses in accord with the histological differences among STSs, phase 3 clinical trials of new drugs targeting patients who are refractory to doxorubicin have begun to enroll STS patients with limited histological subtypes. In the 2010’s, three new drugs for STS were approved based on the results of phase 3 trials, but the histological subtypes of the patients enrolled in the trials of each drug differ. The enrollment or exclusion of major histological subtypes in each phase 3 trial are summarized in Table 2.

The Palette trial, a phase 3 trial of pazopanib compared to placebo for STS patients, excluded liposarcoma based on the preceding phase 2 trial, EORTC 62043 [25]. That phase 2 trial allocated STS patients to four cohorts by their histological subtypes; leiomyosarcoma, liposarcoma, synovial sarcoma, and other subtypes. The efficacy of pazopanib was evaluated on a cohort-by-cohort basis, and of the four cohorts, only the liposarcoma cohort failed to achieve the primary end point. Pazopanib is now approved for STS based on the results of the Palette trial, but in many countries liposarcoma is excluded from the indications for pazopanib.

Trabectedin was approved in Europe in 2007 for the treatment of STS without a phase 3 trial, and it is prescribed in daily practice regardless of the histological subtypes. There are plenty of retrospective clinical data regarding the use of trabectedin for STS. The accumulated retrospective clinical reports revealed that trabectedin treatment showed relatively high clinical efficacy in leiomyosarcoma and liposarcoma [26,27]. Thus, a phase 3 trial of trabectedin compared to dacarbazine was designed to enroll only patients with leiomyosarcoma or liposarcoma (collectively referring to the patients as ‘L-sarcoma’). The results of the trial demonstrated the prolongation of the progression-free survival (PFS) of the L-sarcoma patients treated with trabectedin [23], and these results were the basis of the FDA approval of trabectedin for treating L-sarcoma [28]. Clinical data from European clinical practices also suggested that trabectedin could be beneficial for STS patients with chromosomal translocation, represented by myxoid liposarcoma [29,30]. After these data were obtained, randomized clinical trials evaluated the efficacy of trabectedin for only STS patients with chromosomal translocation [31,32].

Eribulin as a treatment drug for STS started as a phase 2 trial that incorporated various major STS histological subtypes and evaluated the efficacy in each cohort [33]; the exploratory process was similar to that of pazopanib described above. The results of a phase 2 trial suggested that eribulin was effective against leiomyosarcoma and liposarcoma (L-sarcoma), and thus the phase 3 trial was designed to enroll these two histological subtypes. In that trial, eribulin prolonged the patients’ OS compared to dacarbazine [24]. However, a subanalysis of the trial’s data revealed that the OS benefit provided by eribulin was remarkable in the liposarcoma patients but not in the leiomyosarcoma patients [34,35]; as the result, FDA approved eribulin only for liposarcoma [36].

These new drugs (i.e., pazopanib, trabectedin and eribulin) were approved to treat STS based on the results of phase 3 trials to which patients of specific histological subtypes were allocated. The criteria regarding the histological subtypes to be enrolled in the phase 3 trials were decided based on preceding phase 2 trials or retrospective clinical data, but there were limited responses to the drugs among the specific histological subtypes, based on the mechanisms of the antitumor drug or tumor progression. Some clinical reports and prospective trials described liposarcoma patients’ response to pazopanib and non-liposarcoma patients’ response to eribulin [37,38].

The lack of information about the preclinical mechanisms of patient response and tumor progression and the limited basic science data might cause mistakes in histology-targeted treatment strategies. For example, the recent randomized clinical trial ISG-STS 1001—which compared the efficacy of the standard preoperative chemotherapy (a combination of anthracycline and ifosfamide) to ‘histology-tailored’ chemotherapies—failed to show the clinical benefit of the histology-tailored chemotherapies [39]. In the ISG-STS 1001 trial, the histology-tailored regimens consisted of trabectedin alone for the myxoid liposarcomas, a combination of gemcitabine and dacarbazine for the leiomyosarcomas, high-dose ifosfamide for the synovial sarcomas, a combination of etoposide and ifosfamide for the malignant peripheral nerve sheath tumors (MPNSTs), and a combination of gemcitabine and docetaxel for the undifferentiated pleomorphic sarcomas (UPSs). These regimens were selected as tailored therapy based on previous clinical findings, but the evidence level of that clinical data was not high, and in most pathological histologies the mechanism of the response of the specific histology to the tailored therapy remained unknown. As a result, as observed in the results of the whole patient population, the subanalysis revealed that the survival of the patients in the tailored therapy cohorts was inferior to that of the cohorts who received standard therapy, except for the myxoid-liposarcoma cohort. Those results also suggested that in a localized, resectable setting, anatomical and surgical factors (e.g., the tumor size, availability of gross resection, and surgical margins) could be more important to the prognosis of STS patients than the choice of chemotherapy regimen.

## 4. Investigating Molecular Targeted Therapies for STS Patients

Molecular targeted drugs began to be investigated in the 2000’s and were approved for the treatment of many malignant diseases, including hematological diseases and solid tumors. One of the molecular targeted drugs that was approved for solid tumors in the early days is imatinib, a tyrosine kinase inhibitor (TKI) targeting c-kit (CD117), which is specifically expressed in gastrointestinal stromal tumors (GISTs) [40].

GIST is a mesenchymal tumor that originates from submucosal tissues in the gastrointestinal tract. It is regarded as one of the STS histological subtypes, but GISTs are known to be resistant to doxorubicin-based chemotherapy, the standard systemic chemotherapy for STSs as discussed above in Section 2 [41]. Based on the knowledge of the expression of c-kit, the treatment target was c-kit in the first GIST patient who received imatinib; the patient showed a remarkable response [42]. The investigation of imatinib as a treatment for GISTs then progressed rapidly, and treatment strategies and the paradigm of GIST became different from those of other STS subtypes [43]. Following imatinib, new TKIs such as sunitinib and regorafenib were investigated and approved to treat GISTs [44,45]. Of them, regorafenib was also investigated to non-GIST STS and showed some clinical benefits in a randomized phase 2 trial [46].

Encouraged by the success of molecular targeted therapies to GIST, research groups have pursued new treatment targets for STS, and the new candidates for molecular targeted therapies include many growth factor receptors that are overexpressed on the membranes of STS, proteins that are thought to regulate a signaling pathway of tumor progression, and fusion genes promoted by chromosomal translocations.

Tumor angiogenesis has been investigated as a target of therapeutic interventions to solid tumors since the 1990’s, and many growth factors associated with tumor angiogenesis were discovered, including members of the vascular endothelial growth factor (VEGF) family and their receptors [47]. Pazopanib, the phase 3 trial of which was discussed above in Section 3, is a TKI that inhibits VEGF receptors as well as other growth factors, and there are retrospective clinical reports that suggested pazopanib treatment resulted in relatively higher response rates in vascular-abundant VEGF-expressing sarcomas compared to other histological subtypes [48]. Patients with an alveolar soft part sarcoma (ASPS), characterized by translocation t(X;17)(p11.2;q25) and known as a vascular-rich tumor, showed high responses to VEGF-targeted therapies in prospective trials, including trials of pazopanib [49,50,51].

Other than TKIs, bevacizumab, a representative VEGF-targeted antibody that is approved for many solid tumors, also produced modest responses in angiosarcoma patients [52]. In randomized trials, however, the VEGF-targeted therapy did not show significant survival prolongation [53,54]; some of the negative results might be due to low statistical power with only small patient populations. The clinical relevance of the expression of VEGF or related factors as biomarkers of pazopanib or other angiogenesis inhibitors is not yet known.

Insulin-like growth factor 1 receptor (IGF1-R) is a growth factor that is expressed with and related to tumor progression in some histological subtypes of STS [55]. High IGF1-R expression was observed in Ewing sarcoma, and this tumor is regarded as a candidate for targeted therapy [56,57]. Preclinical results encouraged the enrollment of STS patients in early-phase clinical trials of IGF1-R inhibitors, one of which is figitumumab, whose clinical efficacy against STSs including Ewing sarcoma was evaluated [58,59]. However, the investigation of figitumumab was terminated without a phase 3 trial of Ewing sarcoma due to the negative results of a phase 3 trial for non-small cell lung cancer [60]. Another IGF1-R inhibitor is ganitumab, whose efficacy against Ewing sarcoma was evaluated [61]. A phase 3 trial of ganitumab added to chemotherapy was performed, but the trial did not show any survival benefit of ganitumab [62].

The phosphatidylinositol 3-kinase (PI3K)/AKT/mammalian target of rapamycin (mTOR) signaling pathway is dysregulated in many malignant diseases, including STS [63]. TKIs that target PI3K/AKT/mTOR have been investigated for treating STS patients. Ridaforolimus, an intravenous mTOR inhibitor, was evaluated for STS treatment, and a phase 3 trial as maintenance therapy (the SUCCEED trial) was performed; the primary endpoint of PFS was met, but the effect size was not considered clinically relevant and the OS in the ridaforolimus arm was not superior to that of the placebo arm [64]. The phase 3 trial of ridaforolimus enrolled STS patients regardless of histological subtypes, which presents the same problem of study design discussed in Section 2.

In selected histological subtypes, the clinical benefit of mTOR inhibitors might be more remarkable. Perivascular epithelioid cell tumor (PEComa), a mesenchymal tumor composed of distinctive perivascular epithelioid cells, is one of the rare histological subtypes of STS. Activation of the mTOR pathway due to TSC1/TSC2 mutation has been observed in PEComa [65]. There are some clinical data regarding the use of sirolimus and other mTOR inhibitors as off-label treatment for PEComas, and the PEComas showed relatively high responses to mTOR inhibitors compared to other approved antitumor drugs; however, there are few data from prospective clinical trials [66].

Murine double minute 2 (MDM2) and cyclin-dependent kinase 4 (CDK4) are both coded on chromosome 12q13-15 and are known to be amplified in well-differentiated and dedifferentiated liposarcoma. MDM2 regulates p53, one of the most common tumor-suppressive genes. CDK4, a member of the cyclin-dependent kinase family, regulates cell cycles [67]. MDM2 and CDK4 were already familiar as important factors in the pathological diagnosis of liposarcoma, but as MDM2- or CDK4-targeted drugs were investigated, their efficacies against STS (especially liposarcomas) were also evaluated. The investigation of MDM2 inhibitors has been limited to preclinical and early-phase trials [68,69], but there are some phase 2 results for CDK4 inhibitors; liposarcomas showed modest responses to them [70,71]. However, the response rates were not high enough to obtain approval for a single-arm phase 2 trial, and further randomized clinical trials are necessary to confirm the clinical efficacies of CDK4 inhibitors.

Exportin-1 (XPO1), which works as a nuclear transport protein, is regarded as a new treatment target in malignant diseases. Selinexor is the first XPO1 inhibitor approved to treat multiple myeloma (in 2019) [72]. Selinexor was suggested to have antitumor activity against liposarcoma in a preclinical study [73], and prospective clinical trials of selinexor are now underway [74,75].

In the 2010’s, immune checkpoint inhibitors changed the treatment paradigms for many malignant diseases, and the responses of STS patients to these inhibitors were also evaluated. STSs were reported to express programmed death ligand-1 (PD-L1) [76,77], which was proposed as a predictive biomarker of immune checkpoint inhibitors in many solid tumors [78]. However, high PD-L1 expression in STSs is not correlates with the clinical response; immune checkpoint inhibitors did not show clinically meaningful benefits in prospective clinical trials [79,80]. The tumor mutation burden (TMB), another biomarker of the response to immune checkpoint inhibitors, is a concept that has been suggested to explain why there are STS patients who respond to immune checkpoint inhibitors to some extent, but STSs are reported to have a low TMB in general [81], and thus it may not be effective to select STS patients who would respond to an immune checkpoint inhibitor based on their TMB. Microsatellite instability (MSI) is an also predictive marker of immune checkpoint inhibitors [82], and pembrolizumab, one of immune checkpoint inhibitor is approved to MSI-high solid tumors agnostic of primary organs [83]. It was reported that the rate of MSI-high patients among STS patients is extremely low [84]. On the other hand, there is an exceptional STS histological subtype immune checkpoint inhibitors are highly effective, i.e., alveolar soft part sarcoma (ASPS) [85,86]. ASPS is characterized by the specific chromosomal translocation t(X;17)(p11.2;q25) and is a vascular-rich sarcoma [87], but the mechanism underlying its response to immune checkpoint inhibitors is unknown; PD-L1 expression, the TMB, and the MSI-high status do not explain the high response rate of ASPS patients to immune checkpoint inhibitors.

There are some positive data from single-arm clinical trials for the molecular targeted therapies discussed above in this section, but most of the existing data are not clinical evidence obtained in randomized clinical trials, due to the rarity of patients with specific histological subtypes that harbor the targets. For many of the molecular targets, the preclinical evidence regarding effects on tumor genesis and progression and high-level clinical evidence that supports the clinical effectiveness of the targeted therapies is lacking. Where such evidence is available, the preclinical expectancy did not correlate with the clinical results; e.g., for the IGF1-R targeted therapy.

Some fusion genes that are structured by chromosomal translocations act as driver mutations in tumorgenesis, and targeting these fusion genes has often resulted in a patient response rate that is high enough to get approval without or before confirmation by randomized clinical trials [88]. Regarding STS, the anaplastic lymphoma kinase (*ALK*) and neurotrophic receptor kinase (*NTRK*) rearranged fusion genes are good examples. Inflammatory myofibroblastic tumor (IMT), which consists of rare components of STS, harbors specific fusion genes including *ALK* [89,90], and TKIs inhibiting ALK showed high responses to ALK-arranged IMT in prospective clinical trials [91]. NTRK rearrangement is observed at a low rate in many solid tumors [92], but in specific STS histological subtypes such as infantile fibrosarcoma, NTRK rearrangements were observed at high rates [93,94]. Solid tumors (regardless of tumor origin) showed high responses to NTRK-targeted TKIs [95,96], and the NTRK-targeted TKIs were approved for the treatment of solid tumors with NTRK rearrangements, including STS.

These prominent and remarkable treatment targets are present in the very rare components of STS, and they cannot be detected by only clinical features. For the investigations and, if approved, the use of targeted therapies, the pathological characteristics of each patient must be identified and evaluated from the standpoint of whether or not the patient has targetable mutations. These evaluations should be continued to histological subtypes to which particular molecular targets has not been shown such as epithelioid sarcoma or solitary fibrous tumor [97,98].

## 5. Whole-Genome Sequencing for Precision Medicine for STS Patients

With the ongoing progress in genome sequence analysis technology, the whole genomes of malignant diseases including STS have been revealed [99,100], and it is now possible to use genomic analyses in practical medicine [3]. The prospective analysis of panel testing by next-generation sequencing (NGS) in Japan (TOP-GEAR) project obtained the gene-profiling data of 42 sarcoma patients (including both STS and bone sarcoma patients), who comprised 22% of 187 patients with solid tumors [101]. The high rates of STS patient enrollment in the prospective panel testing study suggests that there is a great need for comprehensive genome profiling in STS investigations as well as investigations of other solid tumors. The fact that STS includes relatively higher rates of pediatric patients with heterogeneous pathological subtypes than other solid tumors is also the basis of the need [102].

As discussed in Section 4 above however, the availability of genome profiling does not lead directly to new personalized targeted therapies. The MSK-IMPACT study, which is the largest-scale prospective gene profiling study performed to date, evaluated genomic alterations in genes and molecular pathways of more than 10,000 advanced cancer patients, but only 11% of the patients were able to participate in genomically matched clinical trials [103]. ProfiLER trial, another program to select molecular-based recommended therapies for metastatic cancer patients, included 2579 patients but less than 1% of them experienced an objective response [104]. The mutations unveiled by NGS or other genomic testing in STS include diverse and various genomic alterations, but most of them do not have potent targeted therapies, which are yet to be discovered [100,105]. If a potential targeted therapy is identified, the targeted therapy may or may not show clinical benefits—as in the SHIVA trial, which compared various ‘targeted therapies’ to conventional cytotoxic chemotherapies in solid tumors, with no observed prolongation of the patients’ PFS by the targeted therapies [106].

The reasons for that failure might include inappropriate targets, inappropriate mutation types, and/or inappropriate drug design. An example of inappropriate mutation is ALK amplification. In some rhabdomyosarcomas, ALK was shown to be expressed by immunohistochemistry, but ALK expression in a rhabdomyosarcoma is due to amplification and not a fusion gene as discussed in Section 4 [107]. The prospective clinical trial of an ALK inhibitor to treat rhabdomyosarcoma did not show clinical meaningful efficacy [108]. On the other hand, crizotinib, an ALK inhibitor which has been investigated to rhabodmyosarcoma with ALK expression above, is known as inhibiting other targets such as MET, and showed some clinical responses to clear cell sarcomas overexpressing MET [109]. For rare diseases, rare mutation-targeted therapies are pursued, and the importance of detecting targeted mutations precisely (including mutation types) is greater; the risk of misdiagnosis should also be considered [110].

## 6. Conclusions

Antitumor drugs approved for the treatment of STS have been evaluated and approved for almost all of the STS histological subtypes. In the era of precision medicine and genome sequencing, the efficacy of new drugs should be evaluated in two ways: mutation-based, tumor-agnostic targeted therapy or limited to specific histological subtypes characterized by specific mutations. In the investigations of new targeted therapies for rare diseases such as STS, it should be avoided that multiple drugs with similar mechanisms would be evaluated by only small clinical trials respectively, and that none of those drugs could be eventually approved due to lack of high level clinical evidence. To overcome the rarity of STS, the collaboration of physicians, healthcare institutions, industry and academic centers is necessary [111].

As genome profiling becomes more widely available and common, the genome evaluation data of STS and other rare diseases could be obtained in a unified style, and registration systems of STS (such as a registry) should be created to include genome data. Recent registration systems of cancer patients have obtained useful clinical data that can be used to evaluate patient prognoses, treatment modalities, and drugs [112]. With the inclusion of genomic analysis data in these registration systems, the accumulation of ‘big data’ could accelerate the investigations and efficient enrollment of patients for targeted therapy studies, regardless of the need for clinical trials or clinical practice data.

Clinical trial designs must also be optimized; umbrella trials and basket trials have been advocated as new clinical trial designs to incorporate and evaluate rare fraction and rare tumors [113]. These designs could enable patient enrollments and evaluations of rare malignant diseases such as STS in studies of treatment efficacy, and the designs could become the basis of approvals for new drugs that are agonistic of primary origin [82,95,96].

## Figures and Tables

**Table 1:** Phase 3 clinical trials comparing doxorubicin combination with another antitumor drug to doxorubicin monotherapy in STS patients.

Trial Name	Antitumor Drug Adding to Doxorubicin ^a^	Median OS (Months)	Hazard Ratio [95%CI]	*p*-Value	Ref.
EORTC 62012	Ifosfamide	14.3 vs. 12.8	0.83 [0.67, 1.03]	0.076	[6]
PICASSO III	Palifosfamide	15.9 vs. 16.9	1.05 [0.79, 1.39]	0.74	[7]
SARC021	Evofosfamide	18.4 vs. 19.0	1.06 [0.88, 1.29]	0.527	[8]
ANNOUNCE	Olaratumab	20.4 vs. 19.8	1.05 [0.84, 1.30]	0.69	[9]

^a^ In all trials, the control arm treatment was doxorubicin 75 mg/m^2^ per 3 weeks.

**Table 2:** Histological subtypes of the STS patients enrolled and excluded in phase 3 clinical trials of late-line chemotherapy.

Antitumor Drug	Enrollment by Histological Subtype				Ref.
	Leiomyo-Sarcoma	Liposarcoma	Synovial Sarcoma	Undifferentiated Pleomorphic Sarcoma	
Pazopanib	〇	×	〇	〇	[22]
Trabectedin	〇	〇	×	×	[23]
Eribulin	〇	〇	×	×	[24]

## Abbreviations

ALK	anaplastic lymphoma kinase
ASPS	alveolar soft part sarcoma
CDK4	cyclin-dependent kinase 4
EMA	European Medicines Agency
EORTC	European Organisation for Research and Treatment of Cancer
FDA	Food and Drug Administration
GIST	gastrointestinal stromal tumor
IGF1-R	insulin-like growth factor 1 receptor
IMT	inflammatory myofibroblastic tumor
MDM2	murine double minute 2
MSI	microsatellite instability
MPNST	malignant peripheral nerve sheath tumor
mTOR	mammalian target of rapamycin
NGS	next generation sequencing
NTRK	neurotrophic receptor kinase
OS	overall survival
PDGFR	platelet-derived growth factor receptor
PD-L1	programmed death ligand-1
PEComa	perivascular epithelioid cell tumor
PFS	progression-free survival
PI3K	phosphatidylinositol 3-kinase
STS	soft tissue sarcoma
TKI	tyrosine kinase inhibitor
TMB	tumor mutation burden
UPS	undifferentiated pleomorphic sarcoma
VEGF	vascular endothelial growth factor
XPO1	exportin-1
